# Enzymology of Ca^2+^-Mobilizing Second Messengers Derived from NAD: From NAD Glycohydrolases to (Dual) NADPH Oxidases

**DOI:** 10.3390/cells12040675

**Published:** 2023-02-20

**Authors:** Andreas H. Guse

**Affiliations:** The Calcium Signalling Group, Department of Biochemistry and Molecular Cell Biology, University Medical Center Hamburg-Eppendorf, Martinistrasse 52, 20246 Hamburg, Germany; guse@uke.de

**Keywords:** Ca^2+^ signaling, NAD(P), ADPR, cADPR, NAADP, NAD glycohydrolase, (dual) NADPH oxidase

## Abstract

Nicotinamide adenine dinucleotide (NAD) and its 2′-phosphorylated cousin NADP are precursors for the enzymatic formation of the Ca^2+^-mobilizing second messengers adenosine diphosphoribose (ADPR), 2′-deoxy-ADPR, cyclic ADPR, and nicotinic acid adenine dinucleotide phosphate (NAADP). The enzymes involved are either NAD glycohydrolases CD38 or sterile alpha toll/interleukin receptor motif containing-1 (SARM1), or (dual) NADPH oxidases (NOX/DUOX). Enzymatic function(s) are reviewed and physiological role(s) in selected cell systems are discussed.

## 1. Main Text

**Figure 1:**
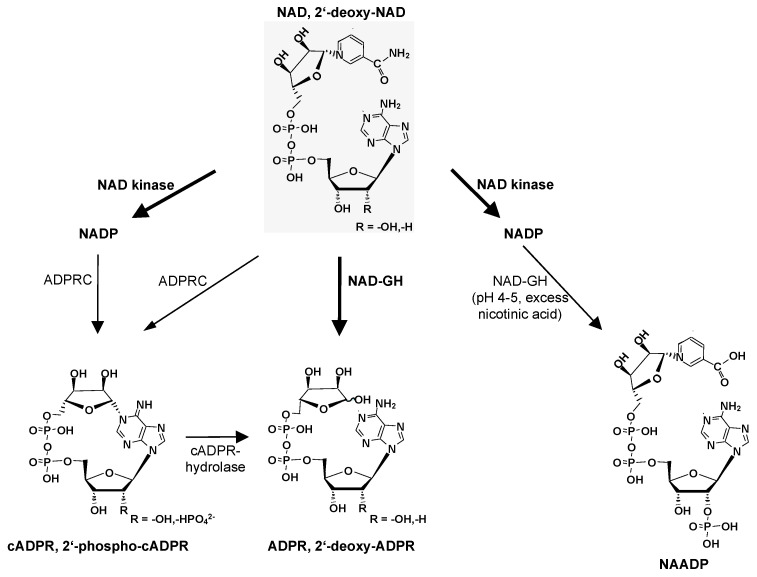
Formation of Ca^2+^-mobilizing second messengers from (2′-deoxy-NAD).

In 1993, Elaine and Mike Jacobson published a seminal paper in *Science* in which they described the role of NAD glycohydrolases (NAD-GHs) in the formation of NAD-derived Ca^2+^-mobilizing second messengers [1]. The authors discovered that a 39 kDa membrane protein metabolized nicotinamide adenine dinucleotide (NAD) to either cyclic adenosine diphosphoribose (cADPR) or to adenosine diphosphoribose (ADPR); these two enzymatic activities carried out by the same enzyme are an ADP-ribosyl cyclase (ADPRC) and a NAD-GH activity (Figure 1). Further, the hydrolysis of cADPR to ADPR by a cADPR-hydrolase activity was also catalyzed by the purified NAD-GH [1] (Figure 1). This was an essential discovery in the fields of both NAD metabolism and Ca^2+^ signaling. Being an active researcher in both these areas, it is a great pleasure for me to contribute to this special issue of *Cells* in honor of Elaine and Mike Jacobson.

While cADPR was identified as a Ca^2+^-mobilizing second messenger through the pioneering work of Hon Cheung Lee in the late 1980s [2,3], ADPR was considered merely an inactive degradation product of NAD in 1993. However, the notion of the 39 kDa membrane NAD-GH as a second-messenger-producing enzyme was revived when ADPR was identified as one of the nucleotides activating the cation channel transient receptor potential, melastatin type 2 (TRPM2) [4].

The above scheme summarizes the formation of ADPR and 2′-deoxy-ADPR through NAD-GH as the main reaction, and the formation of cADPR or 2′-phospho-cADPR via the minor ADPRC activity of NAD-GH. Moreover, the hydrolysis of cADPR by the cADPR-hydrolase activity of NAD-GH is indicated. 

In addition to the synthesis of cADPR and ADPR, there is, at least in the cell-free system, a third enzymatic reaction of the promiscuous 39 kDa membrane NAD-GH, using nicotinamide adenine dinucleotide phosphate (NADP) as a substrate and resulting in the product nicotinic acid adenine dinucleotide phosphate (NAADP) [5] (Figure 1).

In this review, I will start from Elaine’s and Mike’s mechanistic scheme of NAD-GHs and discuss and summarize findings regarding the enzymology of the growing super-family of Ca^2+^-mobilizing second messengers derived from NAD, namely ADPR and its analogue 2′deoxy-ADPR, cADPR and its derivative 2′-phospho-cADPR, and NAADP (Figure 1).

## 2. NAD-GHs

Soon after the *Science* paper published by Elaine and Mike Jacobson [1], the membrane-bound NAD-GH was identified as leukocyte antigen CD38 by Maureen Howard, Hon Cheung Lee, and members of their labs, again a seminal paper that was published in *Science* [6]. Another, more recently discovered NAD-GH is sterile alpha toll/interleukin receptor motif containing-1 (SARM1) [7]. SARM1 catalyzes the same reactions known from CD38, using NAD(P) as substrates. Of note, the two proteins appear structurally unrelated. In the following, the key papers for CD38 and SARM1 will be reviewed.

## 3. CD38

CD38 was formerly known as the T10 antigen and in 1981 described as an integral membrane protein of human thymocytes [8]. Twelve years later, in 1993, the enzymatic nature of CD38 was identified by the groups of Hon Cheung Lee and Antonio De Flora [6,9]. A few months earlier, in 1993, the production of cADPR and ADPR from NAD on the surface of erythrocytes was observed, suggesting that the NAD-GH involved might be an ectoenzyme [10]. CD38 was then characterized regarding its ability to catalyze cADPR formation from NAD and to hydrolyze cADPR to ADPR [6]. When realizing that the active site of the type II membrane protein CD38 is located in the extracellular space, the authors stated, “*The concept of outer membrane expression by a large proportion of lymphocytes of an enzyme that metabolizes NAD+ presents certain intriguing, unresolved issues. In particular, what is the physiological mechanism for regulating this extracellular enzyme and how does its activation lead to intracellular signaling?*” [6]. This question, later termed the ‘topological paradox of CD38 signaling’ by Antonio De Flora and co-workers (reviewed in [11,12]), led to the discovery that (i) CD38 may act as a transporter for cADPR into the cytosol [13], and, in addition, (ii) that CD38 rapidly undergoes endocytosis upon extracellular addition of NAD and produces cADPR in endocytotic vesicles [14]. Briefly thereafter, connexin 43 was identified to transport NAD either through the plasma membrane into the extracellular space or more likely into endocytotic vesicles [15]. De Flora and co-workers also developed and experimentally established the concept of autocrine and paracrine roles for cADPR [16,17,18,19], which were later confirmed by others [20]. Of note, equilibrative and concentrative nucleoside carriers also transport cADPR into cells, e.g., fibroblasts, myocytes, neurons, hemopoietic progenitor cells, and intestinal stem cells [21,22,23]. Concentrative nucleoside carriers, encoded by *Slc28a1*, *a2*, and *a3* [24], are assumed to play a major role since the Na^+^ symport mechanism allows effective cADPR concentrations to build up inside target cells, even from dilute extracellular cADPR solutions (reviewed in [25]).

After these initial years of research on CD38 as a principal mammalian NAD-GH, the expression, enzymatic activity, and role of CD38 in many different cell types were investigated. Moreover, CD38 has been found as a tumor antigen and as a target for tumor therapy in many different tumor cells, e.g., lymphoma (reviewed in [26]), myeloma (reviewed in [27]), and in solid tumors [28]. Using the search term ‘(CD38) AND (NAD-GH)’ 3280 results are currently found in PubMed (20 November 2022), making it impossible to review all the published work. However, a couple of studies using *Cd38*^−/−^ mice or *Cd38*^−/−^ cells are of interest since the results disentangle the physiological function of CD38.

Hiroshi Okamoto and co-workers created a C57Bl6 *Cd38*^−/−^ mouse and showed (i) that glucose-evoked cADPR formation, Ca^2+^ signaling, and insulin secretion in *Cd38*^−/−^ pancreatic beta cells were decreased [29]; (ii) that, in *Cd38*^−/−^ pancreatic acinar cells, Ca^2+^ signaling in response to stimulation of the muscarinic acetylcholine receptor was diminished [30], and (iii) that aortal contraction in *Cd38*^−/−^ mice evoked by α-adrenergic stimulation was reduced [31], suggesting an important role for CD38 in all these Ca^2+^-dependent processes. Using the same *Cd38*-knockout model, Uh-Hyun Kim and co-workers reported that in comparison to wildtype cells (i) in *Cd38*^−/−^ lymphokine-activated killer cells, interleukin-8 evoked, cADPR-dependent Ca^2+^ signaling and migration were impaired [32,33], and that (ii) the angiotensin-II-induced proliferation of *Cd38*^−/−^ liver stellate cells and synthesis of fibronectin and other extracellular matrix proteins were reduced [34].

Another *Cd38*^−/−^ mouse model on a C57BL/6J background was developed in Maureen Howard’s group [35]. Initially, in this model, it was shown that CD38 is neither involved in hematopoiesis nor in lymphopoiesis [35]. Of note, B-lymphocyte activation, proliferation, and cytokine secretion evoked by CD38 signaling require the ectodomain of CD38, but do not depend on CD38′s NAD-GH activity [36]. Two years later, Frances Lund, who was already involved in producing and characterizing *Cd38*^−/−^ mice [35,36], discovered an innate immune function mechanistically dependent on enzymatically active CD38: the fMLP-induced chemotaxis of neutrophils during bacterial infections, described in a seminal paper published in *Nature Medicine* [37]. Lund and co-workers showed (i) the higher susceptibility of *Cd38*^−/−^ mice to *Streptococcus pneumoniae* infection; (ii) reduced endogenous cADPR content in the spleen, thymus, and bone marrow myeloid cells, but not in other organs, such as the lung, kidney, heart, and brain; (iii) diminished chemotaxis in response to fMLP (but not to interleukin-8); (iv) loss of ADP-ribosyl cyclase activity, and (v) largely decreased Ca^2+^ entry [37]. Of note, in a follow-up publication, it was demonstrated that in addition to cADPR, also ADPR played a pivotal role as an activating ligand of the Ca^2+^-permeable cation channel TRPM2 in neutrophil migration [38]. This important result became possible with the development of the first specific antagonist of TRPM2, 8-Br-ADPR, by Tim Walseth and co-workers: 8-Br-ADPR blocked Ca^2+^ entry and chemotaxis in response to fMLP in neutrophils [38]. Moreover, the fact that ADPR acts as a second messenger that activates TRPM2 resulting in Ca^2+^ entry during neutrophil chemotaxis was an important step to prove that, in addition to cADPR, or perhaps even instead of cADPR, ADPR affects Ca^2+^ signaling through TRPM2 activation in cells in general. This change in view of the importance of the CD38 products towards ADPR is supported by results from the thorough biochemical characterization of CD38 by Francis Schuber and Philippe Deterre, showing that ADPR is the main product of CD38, whereas cADPR represents a minor side product: “*The results obtained here are not compatible with the prevailing model for the mode of action of CD38, according to which this enzyme produces first cyclic ADP-ribose which is then immediately hydrolysed into ADP-ribose (i.e., sequential ADP-ribosyl cyclase and cyclic ADP-ribose hydrolase activities). We show instead that the cyclic metabolite was a reaction product of CD38 rather than an obligatory reaction intermediate during the glycohydrolase activity*” [39]. End-point enzyme assays confirmed that ADPR is the main product of CD38 [40,41,42]. Regarding the physiological role of the nucleotides produced by CD38, it should be noted that in many of the earlier studies TRPM2 was not even known to exist, since it was first described as an ADPR-activated cation channel in 2001 [4]. Obviously, the signaling axis CD38 → ADPR → TRPM2 must be taken into account in all Ca^2+^ signaling studies in which CD38 has been identified as a crucial enzyme, as exemplarily done by Rah and colleagues [43], who demonstrated a crucial role of Ca^2+^ entry through TRPM2 for the polarization of cytolytic granules and degranulation involved in the antitumor activity of NK cells. Along these lines, CD38 was also identified to use 2′-deoxy-NAD as a substrate to synthesize 2′-deoxy-ADPR; this ADPR derivative is much more potent in activation of TRPM2 as compared to ADPR, and is thus termed a TRPM2 superagonist [44].

Another important aspect in CD38 research relates to its orientation within the plasma membrane and also other membranes. As pointed out above, as a type II membrane protein with its active catalytic site in the extracellular space, transport functions for both substrate(s) and product(s) are required for intracellular signaling; this remains true also for CD38 on endocytotic vesicles. However, using specific antibodies against the N-terminus of CD38, it was shown in HL-60 and U937 cell lines, as well as in human primary monocytes, that a small portion of CD38 also exists in the type III orientation [45]. Type III CD38 was shown to be activated through one of the products of NADPH oxidase 4 (NOX4), H_2_O_2_, thereby providing a link between signaling via reactive oxygen species and Ca^2+^ signaling through products of CD38 enzyme activity [46]. Through the specific inhibition of type II CD38 using the covalently bound NAD-GH inhibitor ara-F-NAD [47], and wash-out of excess of the inhibitor followed by permeabilization of plasma membranes, minor residual enzymatic activity of CD38 in type III orientation was also confirmed in Jurkat T cells [44]. These results, although not confirmed in many cell systems, indicate that if a small amount of CD38 in type III orientation exists in a particular cell type, fast formation of the superagonist 2′-deoxy-ADPR or ADPR, followed by TRPM2 activation, is the first downstream pathway to be tested. Whether also cADPR is involved in such pathways needs to be analyzed, e.g., by comparing RYR2 and/or RYR3 knockout models vs. TRPM2 knockouts.

In addition to cADPR, 2′-deoxy-ADPR, and ADPR, there is a fourth second messenger produced by CD38, NAADP (Figure 1). The reaction involved is termed the ‘base-exchange reaction’: NADP is used as a substrate and the reaction proceeds at pH 4 to 5 in the presence of a high excess of nicotinic acid [48], immediately raising the question of whether such conditions would exist in the cellular context. However, endo-lysosomes are intracellular organelles known for their acidic pH (pH 4 to 5) and thus would be an *a priori* niche for the base-exchange reaction to take place. Experimentally, this was tackled by directing CD38 to endo-lysosomes, or by engineering a CD38 variant for lysosomal expression [49]. Endogenous NAADP levels increased under such conditions; the extracellular addition of nicotinic acid further raised NAADP levels [49], indicating that CD38 may produce NAADP in endo-lysosomes. These findings were confirmed later in lymphokine-activated killer cells [50]. In addition, evidence for a physiological role of CD38 in the production of NAADP was also obtained through the knockout of *Cd38* in mouse coronary arterial myocytes stimulated via the Fas receptor [51], lymphokine-activated killer cells stimulated by IL-8 [32], pancreatic acinar cells stimulated by cholecystokinin [52], and mouse cardiac myocytes stimulated via the ß-adrenoceptor [53]. In contrast, in other tissues/cell types, opposing effects of the deletion of *Cd38* were also observed: *Cd38*^−/−^ myometrial cells were not different in terms of their endogenous NAADP levels, neither unstimulated nor stimulated by histamine [54]. Furthermore, tissue samples from the thymus and spleen of *Cd38*^−/−^ mice had similar and somewhat increased endogenous NAADP levels, suggesting that CD38 degrades NAADP to 2′-phospho-ADPR rather than producing this second messenger [55]. Taken together, the role of CD38 in NAADP metabolism remains controversial and, according to the experimental data obtained so far, appears to be variable in different cell types. Novel aspects of NAADP’s enzymology will be discussed below, following the section on SARM1.

## 4. SARM1

The toll/interleukin receptor (TIR) domain has been known as a scaffold protein involved in signaling processes of innate immunity. However, sterile alpha toll/interleukin receptor motif containing-1 (SARM1) was found to posses NAD-GH activity, metabolizing NAD(P) to the products ADPR, cADPR, and NAADP, as detailed above for CD38. In neurons, NAD degradation by SARM1 was first described for TIR domains as a central process resulting in axon degeneration [56,57,58,59,60]. However, NAD-GH activity is also found in TIR domains from archaea, bacteria, and plants, as reviewed in [61].

The enzymology of SARM1 was analyzed in detail by HPLC using recombinantly expressed SARM1 missing the N-terminal mitochondrial localization signal (Figure 4 of reference [62]). NAD was mainly metabolized to ADPR, whereas cADPR was a minor product; comparison of normalized enzyme activities resulted in an approximately 10-fold higher NAD-GH as compared to the ADPRC activity of SARM1 [62]. The cADPR-hydrolase activity was weak for both CD38 and SARM1, amounting to approximately 2–3% of NAD-GH activity. At pH 4.5 and with an excess of nicotinic acid (2.5 mM), both CD38 and SARM1 converted NADP to NAADP; this base-exchange activity reached >80% of the NAD-GH activity of CD38 and approximately 40% of the NAD-GH activity of SARM1 [62]. Recently, the base-exchange activity of SARM1 was also described to proceed at a neutral pH [63]. Taken together, the major differences between SARM1 and CD38 appear to be (i) the much higher production of cADPR by SARM1, and (ii) the formation of NAADP by SARM1 also at a neutral pH. This indicates that SARM1 might be a much better cellular source for cADPR than CD38. Of note, a few earlier papers described the production of cADPR also in CD38 knockout models [37,64] or soluble ADPRC activity was detected [65]. Thus, re-evaluation of these data in light of SARM1′s ADPRC activity appears reasonable.

The activation of SARM1 is triggered by an increase in nicotinamide mononucleotide (NMN), a process that can be mimicked by a membrane-permeable mimetic of NMN, termed CZ-48 [62]. Very recently, the activated state of SARM1 was analyzed by cryo-electron microscopy using a novel nanobody that recognizes activated SARM1 [66]. NMN is a breakdown product of NAD, formed by pyrophosphatase activity. In addition, NMN serves as an intermediate in the biosynthesis of NAD, but requires sufficient activity of nicotinamide mononucleotide adenylyltransferase 2. Whereas NAD at a normal cellular concentration inhibits SARM1 activation, due to the autoinhibitory activity of the ARM domain, a decrease in NAD resulting in NMN formation activates SARM1 enzymatic activity (reviewed in [61]).

Adaptation to axonal damage is of major importance for nervous function. SARM1 plays a central role in this process by orchestrating neurons’ responses, including cytokine release, the induction of oligodendrocyte death, and the cessation of axon regeneration (reviewed in [67]). Further, SARM1 drives the Wallerian degeneration of the distal stump of the injured axon [68] and is involved in the phagocytosis of remnants of the stump. Finally, neighboring neurons that sense the injured neuron’s activity, e.g., cytokine release, start a stress response in which SARM1 is involved, too (reviewed in [67]). The signaling processes related to adaptation to axonal damage range from the disturbed metabolism of NAD, specifically a decrease in the cellular NAD concentration, and Ca^2+^ signaling evoked by the Ca^2+^-mobilizing second messengers produced by SARM1, ADPR, cADPR, and NAADP (see paragraphs above). Since ADPR is the major enzymatic product of SARM1, Ca^2+^ entry operated by its target channel TRPM2 is central for the processes following axonal degeneration. Further, Ca^2+^ release by cADPR and NAADP contributes to these processes (reviewed in [67]). Of note, other signaling mechanisms, including Jun N-terminal kinases, are involved as important players (reviewed in [67]). This complex situation caused by both diverse signaling processes and cell responses is currently beginning to be understood in full detail.

## 5. NAADPH/NAADP Redox Cycle: (Dual) NADPH Oxidases (NOX/DUOX) and Glucose 6-Phosphate Dehydrogenase (G6P-DH)

**Figure 2:**
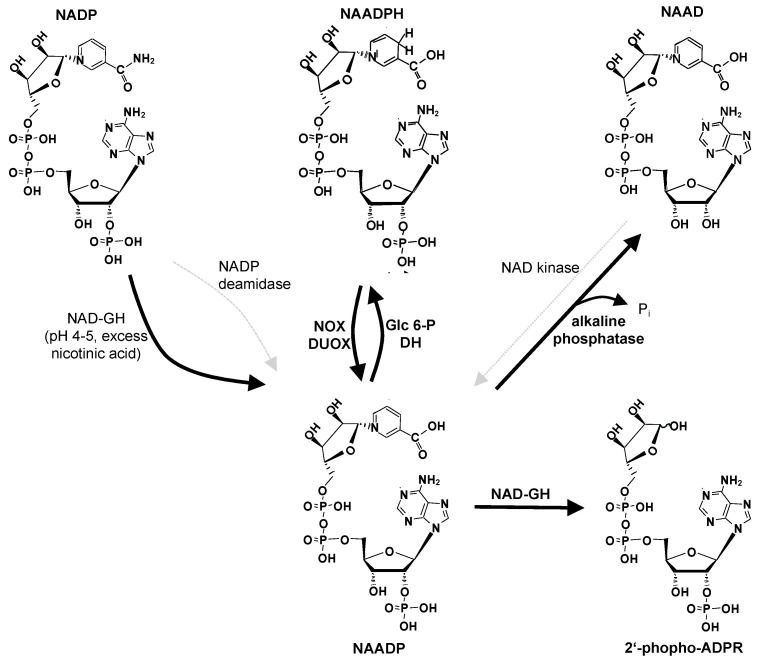
Enzymology of NAADP metabolism: hypothetical and confirmed pathways. Hypothetical pathways are marked by dotted lines in gray. Solid, bold lines indicate confirmed pathways. Abbreviations used: Glc 6-P DH, glucose 6-phosphate dehydrogenase; Pi, inorganic phosphate.

While the cellular formation of ADPR by NAD-GH activity, e.g., catalyzed by CD38 or SARM1, can be regarded as confirmed, the physiological relevance of the base-exchange reaction as a biosynthetic pathway for NAADP is still under debate (Figure 2).

Early on, researchers discussed and experimentally approached alternative pathways. The ‘easiest’ way to produce NAADP from NADP would be an NADP deamidase reaction, whereby the nicotinamide group of NADP is converted to nicotinic acid and NH_4_^+^ leaves the molecule, either as free NH_4_^+^ or by transfer onto an acceptor molecule. Of note, the deamidation of nicotinamide mononucleotide (NMN) to nicotinic acid mononucleotide (NaMN) was previously reported for prokaryotes, e.g., Azotobacter vinelandii [69], Salmonella typhimurium [70], Escherichia coli [71], or Vibrio cholerae [72]. Following these characterizations in single bacterial species, a broadly conserved deamidase (=amidohydrolase; EC 3.5.1.42) enzyme family among prokaryotes was discovered and its gene, termed pncC, cloned by Nadia Raffaelli and colleagues [73]. However, deamidases are only found in prokaryotes. Further, deamidases translated from pncC do not accept NADP as a substrate, and, to the best of my knowledge, other NADP-deamidating enzymes have not yet been published (Figure 2).

Another potential pathway for NAADP production would be NAD kinase. However, NAD kinase accepts NAD as a substrate, but does not phosphorylate nicotinic acid adenine dinucleotide (NAAD) [74] (Figure 2).

A study published by Armando Genazzani’s laboratory showed that NAADP can be reduced to NAADPH by endogenous cellular enzymes, e.g., glucose 6-phosphate dehydrogenase [75]. Further, NAADPH did not release Ca^2+^ in sea urchin egg homogenates [75], suggesting that NAADP’s reduced form, NAADPH, is an inactive derivative that may be converted by an oxidase to NAADP during Ca^2+^ signaling in cells. My own laboratory has been working on the identification of the respective oxidase for a couple of years. Given the fact that NADPH oxidases (NOX; reviewed in [76]) have been known for many years to oxidize the structurally closely related NADPH to NADP, we tested whether NOX5, one of the isozymes of the NOX enzyme family, would oxidize also NAADPH to NAADP. In fact, NAADP was identified in HPLC analysis as a product (Figure 2); this was true not only for NOX5, but also for the isozymes DUOX1 and DUOX2, members of the dual NADPH oxidase subfamily [77]. Enzymatic characterization of NOX5 revealed that both NAADPH and NADPH showed similar Km values and maximal velocities [77]. In contrast to the previous main candidate for NAADP production, CD38, the NOX and DUOX enzymes produce NAADP in the cytosol immediately beneath the plasma membrane, and this enzyme reaction proceeds at an optimal pH of approximately 7.5 [77], only slightly above the cytosolic pH. Since NAADP’s role in the activation of Ca^2+^ microdomains evoked by T cell receptor/CD3 and CD28 stimulation was reported [78], a role for isozymes of the NOX/DUOX family was investigated in this cell system. Regarding expression on mRNA level, mainly NOX1 and NOX2 are expressed in mouse T cells; however, T cells devoid of *Nox1* or *Nox2* showed no Ca^2+^ phenotype upon T cell receptor/CD3 and CD28 stimulation [77]. In contrast, in T cells from *Duoxa1*^−/−^*/Duoxa2*^−/−^ mice, a functional double knockout of DUOX1 and DUOX2 [79,80], both initial Ca^2+^ microdomains as well as global Ca^2+^ signaling were significantly decreased [77]. Single-gene knockouts in rat effector T cells produced by using Crispr/CAS technology revealed a major role of DUOX2 in the first seconds of T cell activation [77]. Of note, deletion of *Duox2* also decreased global Ca^2+^ signaling and specifically decreased IL-17 production upon stimulation of rat effector T cells by antigen-presenting cells [77]. In contrast, in *Cd38*^−/−^ T cells, neither initial Ca^2+^ microdomains nor global Ca^2+^ signaling were affected, when compared to wildtype control T cells [77].

An important aspect to consider when working with NOX/DUOX enzymes is the fact that, in addition to product generation at the cytosolic side of the plasma membrane, e.g., the formation of NAADP, there is also the generation of reactive oxygen species (ROS) at the extracellular side. In the case of DUOX2, H_2_O_2_ is produced in an equimolar concentration to NAADP. Since the cytosolic concentration of NAADP is low—e.g., before T cell receptor/CD3 stimulation, approximately 5 nM, and upon stimulation, approximately 40 nM [81]—it is rather unlikely that the low extracellular H_2_O_2_ concentration would stimulate Ca^2+^ signaling on its own. However, and of note, higher concentrations of H_2_O_2_ have been shown to activate Ca^2+^ entry channels, e.g., TRPM2 [82], and also to inhibit sarcoplasmic and endoplasmic reticular Ca^2+^ ATPase 2 (SERCA2) by sulfonylation [83]. To rule out any effects of the H_2_O_2_ produced during the first few seconds of T cell activation, several control experiments were conducted in the presence of either (i) H_2_O_2_-degrading catalase; (ii) a specific inhibitor of aquaporin3, the H_2_O_2_-transporting aquaporin expressed in T cells; or (iii) the membrane-permeant ROS scavenger butylated hydroxyanisole [77]. None of these interventions had any significant effect on the initial Ca^2+^ microdomains evoked by T cell receptor/CD3 and CD28 stimulation [77]. Furthermore, using HYPER7-MEM for the imaging of potential H_2_O_2_ generation [84] evoked by T cell receptor/CD3 and CD28 stimulation did not result in any significant signals [77].

As already noted by Armando Genazzani and co-workers in 2004, cytosolic dehydrogenases, e.g., ubiquitously expressed glucose 6-phosphate dehydrogenase, can rapidly reduce NAADP to NAADPH [75], thereby providing a novel cytosolic redox cycle for the rapid generation and removal of Ca^2+^-mobilizing NAADP [77] (Figure 2). Since at least some portion of NAADP may also be degraded to 2′-phospho-ADPR by type III CD38, or to NAAD by alkaline phosphatase [85], the NAADPH/NAADP redox cycle likely requires fill-up reactions for NAADPH/NAADP.

Taken together, the NOX/DUOX enzyme family assumes a new role as producers of the highly effective Ca^2+^-mobilizing second messenger NAADP and, in conjunction with glucose 6-phosphate dehydrogenase, constitutes a novel cytosolic redox cycle involved in early local Ca^2+^ signaling, at least in T cells [77].

## 6. Conclusions

NAD(P), for many decades known as a coenzyme of oxidoreductases, is also a precursor for an entire family of Ca^2+^-mobilizing second messengers. The enzymes involved are the NAD-GHs CD38 and SARM1, but also the NOX/DUOX family of NADPH oxidases. While the former mainly produce ADPR under cytosolic conditions, thereby activating the plasma membrane cation channel TRPM2, NOX/DUOX enzymes, at least NOX5, DUOX1, and DUOX2, produce NAADP from its reduced form, NAADPH. Regarding the production of the second messenger cADPR, SARM1 appears to generate more cADPR as compared to CD38.

CD38 and the NOX/DUOX enzymes are regulated by phosphorylation and/or elevated Ca^2+^ concentrations, whereas SARM1 is regulated by the cellular (cytosolic?) ratio of NAD to NMN. Thus, the NOX/DUOX enzymes and CD38 conform to other second-messenger-generating enzymes, e.g., phospholipase C, known to be activated via plasma membrane receptors for hormones, paracrine mediators, neurotransmitters, etc. In contrast, SARM1 seems to sense NAD loss, e.g., due to infection by certain viruses, leading to SARM1 activation followed by more NAD degradation, and finally resulting in cell death. The latter is required to stop viral spread in the body. This situation raises many questions, e.g., can a cell distinguish between ADPR produced by CD38 and ADPR produced by SARM1? Is it a question of potentially different cytosolic ADPR concentrations in response to CD38 and SARM1 activation, or is the higher co-production of cADPR and possibly also NAADP, in the case of SARM1, involved in determining the cell’s fate?

Although many aspects have been clarified since the initial discovery of NAD-GH’s enzymatic function by Mike and Elaine Jacobson in 1993 [1], many open questions remain for the next generation of researchers in the adenine nucleotide and Ca^2+^ signaling field.

## Data Availability

Not applicable.

## Abbreviations

ADPR, adenosine diphosphoribose; ADPRC, ADP-ribosyl cyclase; cADPR, cyclic adenosine diphosphoribose; DUOX, dual NADPH oxidase; NAADP, nicotinic acid adenine dinucleotide phosphate; NAD(P), nicotinamide adenine dinucleotide (phosphate); NAD-GH, NAD glycohydrolase; NAAD, nicotinic acid adenine dinucleotide; NaMN, nicotinic acid mononucleotide; NMN, nicotinamide mononucleotide; NOX, NADPH oxidase; ROS, reactive oxygen species; SARM1, sterile alpha toll/interleukin receptor motif containing-1; SERCA2, sarcoplasmic and endoplasmic reticular Ca^2+^ ATPase 2; TRPM2, transient receptor potential, melastatin type 2.
